# Endophytic Microbes Are Tools to Increase Tolerance in *Jasione* Plants Against Arsenic Stress

**DOI:** 10.3389/fmicb.2021.664271

**Published:** 2021-10-06

**Authors:** Natalia González-Benítez, Irene Martín-Rodríguez, Isabel Cuesta, Manuel Arrayás, James Francis White, María Carmen Molina

**Affiliations:** ^1^Department of Biology, Geology, Physics, and Inorganic Chemistry, Universidad Rey Juan Carlos, Madrid, Spain; ^2^Unidad de Bioinformática, Instituto de Salud Carlos III, Madrid, Spain; ^3^Área de Electromagnetismo, Universidad Rey Juan Carlos, Madrid, Spain; ^4^Department of Plant Biology, Rutgers University, New Brunswick, NJ, United States

**Keywords:** arsenic, gnotobiont, *Jasione sessiliflora*, vertical transmission, horizontal transmission, bacterial endophyte core, *Rhodococcus rhodochrous*, *Jasione montana*

## Abstract

Seed microbiota is becoming an emergent area of research. Host plant microbial diversity is increasingly well described, yet relatively little is known about the stressors driving plant endomicrobiota at the metaorganism level. The present work examines the role of horizontal and vertical transmission of bacterial microbiota in response to abiotic stress generated by arsenic. Horizontal transmission is achieved by bioaugmentation with the endophyte *Rhodococcus rhodochrous*, while vertical transmission comes *via* maternal inheritance from seeds. To achieve this goal, all experiments were conducted with two *Jasione* species. *J. montana* is tolerant to arsenic (As), whereas *J. sessiliflora*, being phylogenetically close to *J. montana*, was not previously described as As tolerant. The *Jasione* core bacterial endophytes are composed of genera *Pseudomonas*, *Ralstonia*, *Undibacterium*, *Cutibacterium*, and *Kocuria* and family *Comamanadaceae* across different environmental conditions. All these operational taxonomic units (OTUs) coexisted from seeds to the development of the seedling, independently of As stress, or bioaugmentation treatment and *Jasione* species. *R. rhodochrous* colonized efficiently both species, driving the endomicrobiota structure of *Jasione* with a stronger effect than As stress. Despite the fact that most of the OTUs identified inside *Jasione* seeds and seedlings belonged to rare microbiota, they represent a large bacterial reservoir offering important physiological and ecological traits to the host. *Jasione* traits co-regulated with *R. rhodochrous*, and the associated microbiota improved the host response to As stress. NGS-Illumina tools provided further knowledge about the ecological and functional roles of plant endophytes.

## Introduction

Microorganisms with 3 billion years of evolution have acquired a wide range of metabolic activities, being able to colonize almost all ecological niches including plants and animals. Plant microbiomes can modify the genomic and metabolic functions of their hosts, improving functions and stress tolerance (Cordovez et al., 2019). In this sense, plants are constituted by the genome of the plant and its associated microbiota, which establish an inter- and intracellular association (Thijs et al., 2016; Cordovez et al., 2019). Plants have a set of microorganisms necessary for their survival considered as core or obligate microbiota (Hardoim et al., 2008; Vandenkoornhuyse et al., 2015; Trivedi et al., 2020), generally obtained by a vertical maternal route (Truyens et al., 2015; Klaedtke et al., 2016; Moreira et al., 2021). Another set of microorganisms is considered accessory or facultative, providing mutualistic or antagonistic interactions to the plant under certain environmental conditions, generally obtained by horizontal transmission (Clay, 1998; Hardoim et al., 2015). Among microbiota associated with host plants, endophytes are “all living organisms inside plant tissues” (De Barry, 1866), but since its origin, this term has evolved over time to become more complex. Some authors have considered this term to refer to the location of the microorganisms (Schulz and Boyle, 2005; Hardoim et al., 2015), but others have included the nature of the interaction with the plant (Perotti, 1926; Wennstrom, 1994; Verma and White, 2019). The lack of consensus on this term may lead to controversy.

The same plant species may recruit different endophyte assemblages depending on the environment. The soil reservoir of endophytes will offer a wide range of physiological functions to the host plant providing growth promotion (Compant et al., 2021), more protection against pathogens (Matsumoto et al., 2021) and herbivory (Verma et al., 2017), and a better adaptation to environmental conditions and abiotic stress (Rodríguez et al., 2008).

Arsenic is considered one of the major stresses to plants (Kumar and Verma, 2018) and is a highly toxic metalloid from anthropogenic and natural sources. Plant exposure to As, even at low concentrations, could trigger negative consequences in morphology, physiology, and biochemistry (Moreno-Jiménez et al., 2008; Abbas et al., 2018). However, some plants are able to complete their life cycles in environments highly contaminated with As (García-Salgado et al., 2011; Guterres et al., 2019; Calabrese and Agathokleous, 2021).

Many studies examine host plant microbial diversity, but studies on the mechanisms of how stressors drive plant endomicrobiota at the metaorganism level are lacking. We hypothesized that endophytes play an important role on host traits against environmental stress. To test this hypothesis, the present work addresses the role of horizontal and vertical transmission of bacteria in two species of *Jasione* against As stress. The choice of this host plant was inclined toward *Jasione montana* L. because it is tolerant to arsenic (Gutiérrez-Ginés et al., 2015; Molina et al., 2021) and *Jasione sessiliflora Boiss. & Reut.*, which is phylogenetically close (sister group) to *J. montana* (Pérez-Espona et al., 2005) and has not been previously described as As tolerant. For bioaugmentation experiments, we used *Rhodococcus rhodochrous*, an As-resistant and plant growth-promoting bacteria endophyte from *J. montana* collected from highly As-contaminated soils (Molina et al., 2021).

## Materials and Methods

### *Jasione montana* and *Jasione sessiliflora*

Seeds of *J. montana* were collected by Pauthier and Lang, 2010 in Saint-Georges (France). The area was located on a road edge in acid rocky ground at 787 m altitude in the Garabit viaduct (N 44° 58′ 21.9″, E 3° 10′ 27.8″). Seeds of *J. sessiliflora* were collected by Pauthier, 1996 in siliceous soils at 650 m altitude in Villefort, Lozère (N 44° 29′ 7″, E 3° 55’25″). Seeds from both species were given to us by the germplasm bank of the French National Museum of Natural History. Seeds were stored in airtight containers at low temperatures (−18°C) after desiccation in desiccation chambers (15% relative humidity).

### Bacteria for Bioaugmentation

*Rhodococcus rhodochrous* (*Nocardiaceae*) is an endophyte isolated from an adult plant of *J. montana* collected in a highly polluted area in the Mónica mine, Bustarviejo (19 × 10^3^ mg kg^–1^). *R. rhodococcus* has been previously identified (Molina et al., 2021) and shows resistance up to 450 mM of arsenate a, produces indole acetic acid (IAA), and inhibits 40% of the growth of *Alternaria* sp., which is a potential pathogenic ascomycete fungus (Soares et al., 2016) also isolated from *Jasione* seeds (Molina et al., 2021). Due to these characteristics, this bacterium has been used for the study the horizontal transmission of bacteria.

### Experimental Design

#### The Role of Endomicrobiota for Plant Development

**FIGURE 1:**
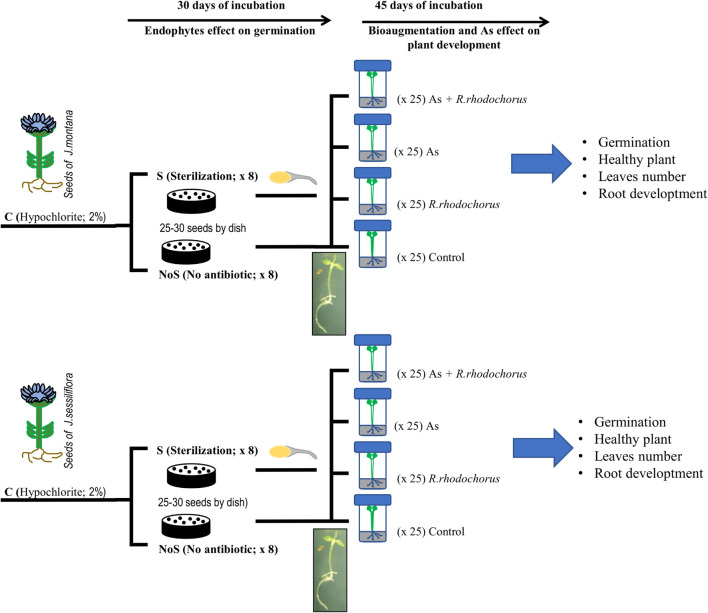
Diagram of the experimental design.

Seeds were subjected to two procedures (Figure 1). Sterilization (S) and No sterilization (no S). For No S, seeds were washed to remove only bacterial and fungal epiphytes with 2% (v/v) sodium hypochlorite during 5 min with slow rotation and rinsed three times with milliQ water (Molina et al., 2021). For the S procedure, seeds were washed with streptomycin sulfate (100 μg/ml, Sigma-Aldrich, MO, United States) during 24 h in continuous agitation (Molina et al., 2021) to obtain a gnotobiotic organism.

Each treatment for each plant was replicated eight times; each replicate consisted of 25–30 seeds per Petri dish in a basal germination medium (0.7% agarose, Sigma-Aldrich). Dishes were incubated in a cultivation chamber (Selecta HOTCOLD-GL, Refrigerated Precision Cabinet) for 30 days at 24°C and 12-h photoperiod cycles with continuous illumination. Dishes were periodically changed to eliminate any fungal contamination. Germination was indicated when seeds showed hypocotyl and/or radicle. Complete development of healthy seedlings was indicated when seedlings had, at least, hypocotyl, main root or radicle, and cotyledons.

#### The Role of Vertical and Horizontal Transmission of Microbiota in Response to as Stress

After 30 days of incubation, we applied four treatments to each of the seeds from C treatment (Figure 1). As treatment (addition of 125μM As[V]; As), bioaugmentation treatment (addition of *R. rhodochrous*; BA), combination treatment (As + BAs), and control treatment (no As + no BA). All experiments were carried out under sterile conditions avoiding any contamination by bacteria except for the controlled colonization by *R. rhodochrous.* All seedlings were planted in autoclaved individual tubes with Murashige & Skoog nutrient medium (Sigma Aldrich). Tubes were cultivated in the same cultivation chamber to have the same temperature and light conditions during germination experiments. We rotated tubes every 2 to 3 days to avoid position effects during the 45 days of incubation. There were a total of 25 seedling per treatment (Figure 1).

The number of leaves and roots and the length of stem and main root were analyzed with the software ImageJ^1^. The main root was measured from the first lateral branch to the root cap. The shoot was measured from the first branch of the root to the apical shoot tip. A plant was considered dead when, visibly, it did not have photosynthetic pigments.

#### Identification of Endophytic Bacteria From Seeds and Seedlings of *J. montana* and *J. sessiliflora*

Seeds (50 seeds from each plant species) and seedlings (four from each plant species) were washed with hypochlorite 2% (v/v) to remove epiphytic microorganisms as we mentioned above. We used DNEASY Plant Kit (Qiagen, Hilden, Germany), and for the seeds, we used DNA Isolation Kit (MO BIO Technologies, CA, United States). Quant-IT PicoGreen reagent (Thermo Fisher Scientific, MA, United States) was used to measure the DNA concentration of each sample. DNA samples were used to amplify the V5–V6 region of the 16S rRNA gene using the protocol in Molina et al. (2021). Also, blocking primers were used in order not to amplify DNA from mitochondria and chloroplast (Arenz et al., 2015). PCR products had a length of approximately 421 bp. PCR products with the extension tails (PE1-CS1 and PE2-BC-CS2) allowed the addition of 10 nucleotide barcodes and a specific Illumina sequence in the second low-cycle number PCR, using a Fluidigm protocol:

V5-F: ACACTGACGACATGGTTCTACAGGATTAGATA CCCTGGTA
V6-R: TACGGTAGCAGAGACTTGGTCTCGACRRCCAT GCANCACCT

PE1-CS1: 5′-AATGATACGGCGACCACCGAGATCTACA CTGACGACATGGTTCTACA-3′
PE2-BC-CS2: 5′-CAAGCAGAAGACGGCATACGAGAT-[10 nucleotides barcode]-TACGGTAGCAGAGACTT GGTCT-3′.

DNA samples were sequenced with Illumina MiSeq (2 × 301). Pass filter reads were prepared using standard procedures (MiSeq Real Time Analysis, Illumina). Sequences were de-noised, merged, and clustered into operational taxonomic units (OTUs) using DADA2 (Callahan et al., 2016) in Qiime 2 version 2020.6 (Boylen et al., 2019). OTUs were aligned and classified with a Qiime2 q2-feature-classifier with a pre-trained Naïve Bayes classifier on the Silva 99% OTUs full-length sequences (version 138)^2^. The number of sequences per sample was rarefied to ensure even sampling (Suplementary Figure 1). Taxonomic assignments were made by comparison with their homolog in GenBank using BLAST (Altschul et al., 1997).

Diversity indices were then calculated on relative abundance OTU tables. Seeds and plant taxonomic richness were expressed as the number of OTUs. The diversity was measured by Simpson’s diversity index (1-Ds), while evenness was measured by *Pielou*’s evenness *index* (J′).

### Statistical Analysis

All data were analyzed with R v.3.6.0 (R Core Team, 2019). We compared germination and plant development among treatments by generalized linear mixed models (GLMMs), including species and treatment as a fixed factor and dish and seed as random factors. Random factors were not significant; therefore, we analyzed germination, plant development, and number of leaves and roots with generalized linear models (GLMs). Data presented heteroscedasticity; therefore, GLMs were estimated with a Poisson distribution for germination, plant development, and number of leaves and roots, but the length of stem and main root was estimated with Gaussian distribution. The best model selection was based on the Akaike’s information criterion (AIC) corrected for small sample size (AICc). Akaike weights (Burnham and Anderson, 2002) were calculated for the final model, including all significant variables, and then for models in which the variables were sequentially excluded.

The similarity among whole samples was estimated with the index similarity coefficient using the Bray–Curtis distance with the function vegdist in the R package Vegan (Oksanen, 2016). The dendrogram was generated using the unweighted pair group with arithmetic means (UPGMA) using the function hclust of the stats package in R (version 1.2.5033). The overlapping areas of the Venn diagrams are used to represent shared OTUs between all treatments (BA, no BA, As, and no As) and species (*J. montana* and *J. sessiliflora*).

## Results

### The Role of *Jasione* Endomicrobiota in Modulating Plant Development

**FIGURE 2:**
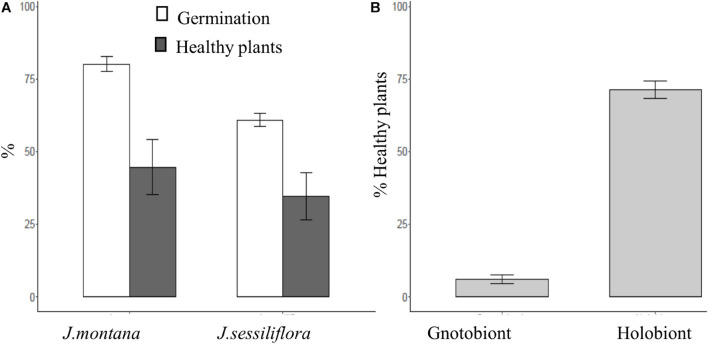
**(A)** Percentage of germination and healthy plants of *J. montana* and *J. sessiliflora* (white and gray, respectively). **(B)** Percentage of healthy plants in gnotobiotic (seeds without endophytes) and holobiotic (seeds with endophytes) models. All statistical parameters are shown in Table 2. The error bars are standard error.

*Jasione montana* (Figure 2A and Table 1) had a significant higher percentage of germination (80.1% ± 2.5%) and healthy plant development (44.7% ± 9.4%) than *J. sessiliflora* (60.9% ± 2.3% and 34.7% ± 8.0%, respectively). The role of *Jasione* endomicrobiota was found to be important for plant development with significantly higher scores (Figure 2B and Table 1) observed in the holobiotic (plant with bacteria endomicrobiota; 71.4% ± 3.0%) than in the gnotobiotic model (6.2% ± 1.5%).

**TABLE 1:** Generalized linear model analyses to test the effect of species (spp.; *J. montana* and *J. sessiliflora*) and sterilization treatment (no S = holobiotic vs. S = gnotobiotic) and interactions of both (sp and sterilization) on the germination and plant development of healthy plants.

Factor	Model	df	AIC	Δ AIC
Germination	Null	1	1015.75	35.45
	**Sp**	**2**	**980.31**	**0.00**
	Sterilization	2	1016.95	36.65
	Sp + Sterlization	3	981.27	0.96
	Sp:Sterilization	4	983.25	2.94

**Factor**	**Model**	**df**	**AIC**	****Δ** AIC**

Healthy plant	Null	1	1115.26	428.39
	Sp	2	1107.10	420.23
	Sterilization	2	707.17	20.30
	**Sp + Sterlization**	**3**	**686.87**	**0.00**
	Sp:Sterilization	4	688.87	2.00
	Sp*Sterilization	4	688.87	2.00

*Null effect as an alternative hypothesis, using the Akaike Information Criterion (AIC). The bold type indicates the best models selected.*

*Test the pure effects of the two factors and the interaction.

*^+^Test only the pure effects of the two factors.*

*:Test only the interaction of the two factors.*

**TABLE 2:** Generalized linear model analyses to test the effect of species (*J. montana* and *J. sessiliflora*), As, and BA (*R. rhodochrous*) on the number of plant leaves and lateral roots.

Factor	Model	df	AIC	Δ AIC
Number of leaves	Null	1	553.872	72.82
	**Sp: As + Sp: BA**	6	481.05	0
	Sp*As + Sp * BA	6	481.05	0

**Factor**	**Model**	**df**	**AIC**	****Δ** AIC**

Number of roots	Null	1	263.49	13.12
	Sp * BA	4	251.71	1.34
	**BA: Sp + BA: As**	6	250.36	0
	BA* Sp + BA*As	8	250.36	0

*Null effect as an alternative hypothesis, using the Akaike Information Criterion (AIC) and difference in AIC score between the best model and the model being compared (ΔAIC). The bold type indicates the best models selected. It shows null model and best model selected.*

*Test the pure effects of the two factors and the interaction.

*^+^Test only the pure effects of the two factors.*

*:Test only the interaction of the two factors.*

### Effects of Arsenic and BA on *Jasione* Develpment

**FIGURE 3:**
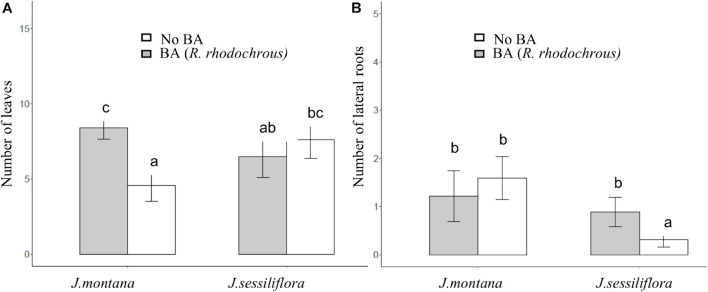
The effect of BA treatment and species of *Jasione* (*J. montana* and *J. sessiliflora*) on the number of leaves **(A)** and the number of lateral roots **(B)**. All statistical parameters are shown in Table 2. The error bars are standard error. Letters indicate the different group of means in SNK tests at *p* < 0.05.

**FIGURE 4:**
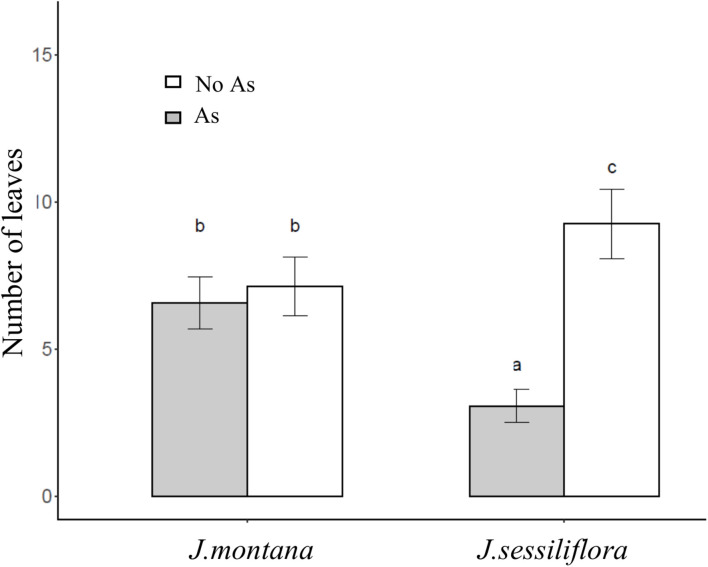
The effect of the As treatment and *Jasione* sp. on the number of leaves. All statistical parameters are shown in Table 2. The error bars are standard error. Letters indicate the different group of means in SNK tests at *p* < 0.05.

**FIGURE 5:**
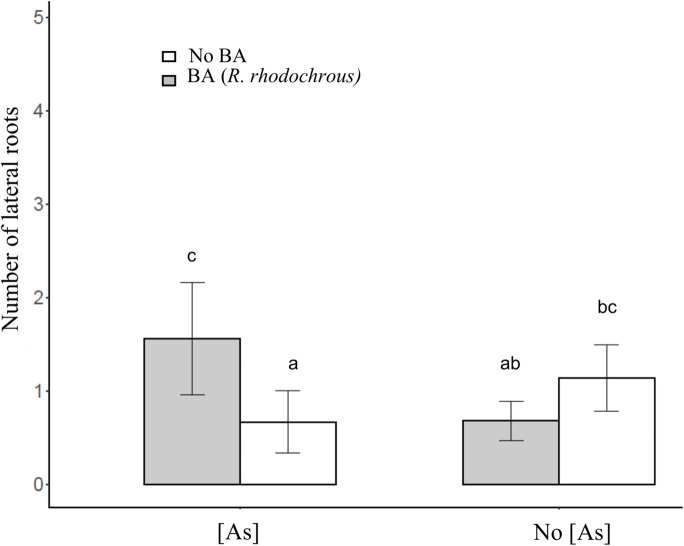
The effect of the BA and As treatment on the number of lateral roots. All statistical parameters are shown in Table 2. The error bars are standard error. Letters indicate the different group of means in SNK tests at *p* < 0.05.

After 45 days of incubation, the treatment BA (addition of *R. rhodochrous*) did not show any effect on the number of leaves in *J. sessiliflora* (7.6 ± 1.2 and 6.5 ± 1.4 leaves without BA and with BA, respectively) whereas it had a positive effect (4.6 ± 1.0 and 8.4 ± 0.8 leaves without BA and with BA, respectively) in *J. montana* (Figure 3A and Table 2). The opposite pattern was observed on the number of lateral roots (LR). The addition of *R. rhodochrous* (treatment BA) did not show any effect in *J. montana* (1.2 ± 0.5 and 1.6 ± 0.4 LR without BA and with BA, respectively) whereas *J. sessiliflora* (0.9 ± 0.3 and 0.3 ± 0.2 LR without BA and with BA, respectively) showed a significantly reduced number of leaves (Figure 3B and Table 2). Accordingly, As did not show any significant effect on the number of leaves (7.1 ± 1.0 and 6.6 ± 0.9 leaves without As and with As, respectively) of *J. montana* (Figure 4 and Table 2) whereas *J. sessiliflora* showed a significantly reduced number of leaves under As stress (9.3 ± 1.2 and 3.1 ± 0.6 leaves without As and with As, respectively). However, the interaction of As and BA treatments was significant (Table 2). The addition of *R. rhodochrous* (BA treatment) increased the number of LR under As stress (0.7 ± 0.3 and 1.6 ± 0.6 LR without BA and with BA, respectively) whereas it did not show any effect (1.1 ± 0.3 and 0.7 ± 0.2 LR without BA and with BA, respectively) under conditions without stress (Figure 5 and Table 2). The lengths of the roots and shoots were not significantly changed in any treatment.

### Molecular Identification and Characterization of *Jasione* Endomicrobiota

**FIGURE 6:**
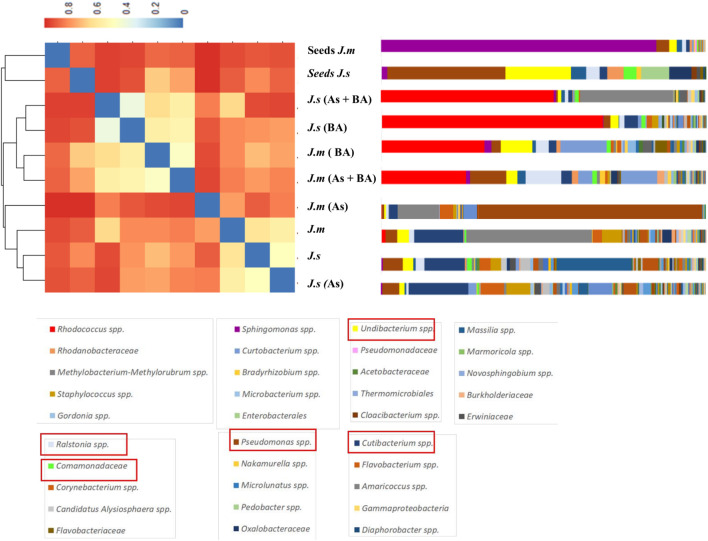
Hierarchical Clustering Heatmap Plot of dendrograms using Unweighted Pair Group Method with Arithmetic Mean (UPGMA) method. The heatmap represents a grid of colored points where each color represents a gradient of dissimilarity. Seeds of *J. montana* (*J. m* seeds), seeds of *J. sessiliflora* (*J. s* seeds), seedlings of *J. montana* with BA (*R. rhodochrous*) and As (*J. m* + BA + As), seedlings of *J. sessiliflora* with BA and As (*J. s* + BA + As), seedlings of *J. montana* with BA (*J. m* + BA), seedlings of *J. sessiliflora* with BA (*J. s* + BA), seedlings of *J. montana* (*J. m*), *s*eedlings of *J. sessiliflora* (*J. s*), and seedlings of *J. montana* with As (*J. m* + As).

**FIGURE 7:**
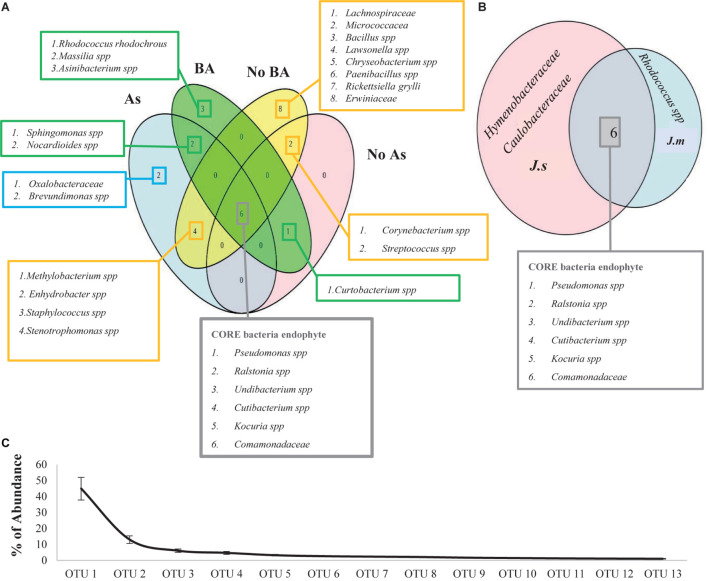
**(A)** The contribution of the OTUs in each group of samples, resulting from Illumina 16S rRNA gene analysis. Seeds of *J. montana* (*J. m* seeds), seeds of *J. sessiliflora* (*J. s* seeds), seedlings of *J. montana* with BA (*R. rhodochrous*) and As (*J. m* + BA + As), seedlings of *J. sessiliflora* with BA and As (*J. s* + BA + As), seedlings of *J. montana* with BA (*J. m* + BA), seedlings of *J. sessiliflora* with BA (*J. s* + BA), seedlings of *J. montana* (*J. m*), *s*eedlings of *J. sessiliflora* (*J. s*), and seedlings of *J. montana* with As (*J. m* + As). The OTUs in red squares belong to the core microbiota of *Jasione.* **(B)** Venn diagram shows all OTUs presented at all *Jasione* samples (Jasione core bacteria endophytes; gray box), OTUs present at all samples BA (green boxes), OTUs present at all samples no BA (yellow boxes), OTUs present at all samples grown under As conditions (blue boxes), and OTUs present at all samples grown without As (red boxes). Venn diagram shows *Jasione* core bacteria endophytes (gray box) and the exclusive core bacteria endophytes to *J. montana* (blue box) and *J. sessiliflora* (red box). **(C)** Average (SE) dynamic rank abundance curve for endophytes within seeds and seedlings of *Jasione* under different treatments SI. Rarefaction curves generated by QIIME reflects the sequencing depth of each sample under a similarity of 0.97. Rarefaction curves were saturated indicating that the sampling depths were sufficient to capture the overall microbial diversity of all samples.

*Rhodococcus rhodochrous* was the dominant OTU when it was inoculated and was present in all adult plants under BA treatments (Figures 6, 7). Cluster dendrogram analysis (UPGMA) based on the relative abundance of all OTUs revealed three major distinct clusters (Figure 6): (1) seeds group, (2) BA group, and (3) NoBA group. Among OTUs identified through high-throughput sequencing, the most common OTUs observed inside *Jasione* samples were genera *Pseudomonas* spp., Ralstonia spp., *Undibacterium* spp., *Cutibacterium* spp., *Kocuria* spp., and *Comamonadaceae* family (Figure 7A). Even though *Jasione* plants were grown under sterile conditions to avoid contamination, the number of OTUs within seeds was lower than those present in adult plants (Table 3). OTUs exclusively associated with plants growing under As stress were genus *Brevundimonas* spp. and family *Oxalobacteraceae*; shared with BA treatments were genera *Nocardioides* spp. and *Sphingomonas* spp.; and shared with NoBA were genera *Methylobacterium* spp., *Enhydrobacter* spp., *Staphylococcus* spp., and *Stenotrophomonas* spp. (Figure 7A). OTUs exclusively associated with plants in the BA treatment included species *R. rhodochrous* (which was inoculated), *Massilia* spp., and *Asinibacterium* spp. OTUs exclusively present in the NoBA treatment were families *Lachnospiraceae*, *Erwiniaceae*, and *Micrococcaceae* and genera *Bacillus* spp., *Lawsonella* spp., *Chryseobacterium* spp., *Paenibacillus* spp., and *Rickettsiella grylli.* No OTUs were exclusively in No As treatment (Figure 7A). The OTU exclusively associated with *J. montana* was *Rhodococcus* spp. and those associated with *J. sessiliflora* were the families *Hymenobacteraceae* and *Caulobacteraceae* (Figure 7B). A dynamic rank-abundance distribution curve for the endomicrobiota of *Jasione* is shown in Figure 7C. A small number of OTUs were dominant, while most of the OTUs were considered rare because of their low abundances.

**TABLE 3:** Average (± SE) and ANOVA for the effects of species of *Jasione*, As, and BA treatment on total number of OTUs (S), Simpson index of diversity (Ds), and *Pielou’s* evenness *index* (J′).

	OTUS		Simpson index of diversity (Ds)		*Pielou*’s evenness *index* (J′)	
*J. montana* *J. sessiliflora*	66 ± 5 72 ± 8	*F*_1,6_ = 0.45; *p* = 0.52	0.77 ± 0.09 0.76 ± 0.10	*F*_1,6_ = 0.005; *p* = 0.9	0.53 ± 0.15 0.44 ± 0.09	*F*_1,6_ = 0.29; *p* = 0.60

As No As	73 ± 2 65 ± 9	*F*_1,6_ = 0.84; *p* = 0.34	0.74 ± 0.10 0.79 ± 0.09	*F*_1,6_ = 0.09; *p* = 0.77	0.40 ± 0.10 0.57 ± 0.13	*F*_1,6_ = 1.07; *p* = 0.33

BA No BA	62 ± 7 76 ± 4	*F*_1,6_ = 3.09; *p* = 0.12	0.73 ± 0.09 0.80 ± 0.10	*F*_1,6_ = 0.28; *p* = 0.61	0.39 ± 0.06 0.58 ± 0.15	*F*_1,6_ = 1.5; *p* = 0.26

Seeds	23 ± 6		0.54 ± 0.27		0.34 ± 0.17	

## Discussion

The study of seed endomicrobiota is an emergent area of research (Nelson, 2018; Verma and White, 2019; Franco-Franklin et al., 2021; Xu et al., 2021). Diversity of seed endomicrobiota is highly underestimated since most studies have used culturable techniques and not culture-independent approaches (Hardoim, 2019). Very little is known regarding how the seed endobiota modulates plant development.

### The Role of Endomicrobiota in *Jasione* Development

*Jasione* seeds contained an average of 23 ± 6 OTUs. During *Jasione* seed imbibition and germination, seed endomicrobiota become activated (including germinating endospores) to create part of the spermosphere and play a role in development of the seedling and the adult plant (Chang et al., 2021; Molina et al., 2021). OTUs found in adult plants (70 ± 6) likely came from seeds (Barret et al., 2015). Seeds are in a dormant state, and it is probable that bacteria in seeds are also in a state of dormancy (Truyens et al., 2015). Since seed endomicrobiota are exposed to high osmotic pressures within seeds, endospore formation may be a feature to withstand this environment (Compant et al., 2011; Truyens et al., 2015). However, standard DNA purification procedures are not suitable to disrupt endospores. Some authors (e.g., Esteban et al., 2020) have proposed, as an alternative approach, a microwave treatment before the standard method to extract DNA from endospore and resistant structures. The lower number of OTUs identified inside the seeds is probably due to the presence of endospores where DNA was not extracted and amplified. Our findings suggest that combined OTU identifications from sterilized seeds and plants developed under axenic conditions show a more complete seed endomicrobiota diversity.

When endomicrobiota were removed from seeds with antibiotics, the gnotobiotic plants show a lower percentage of germination and plant development than holobiotic plants (plants with endomicrobiota associated). Previous studies have suggested that antibiotic treatment might cause irreparable damage to seed organelles, such as mitochondria (Robert and Hevor, 2007). However, the addition of endophytes to gnotobiotic organisms frequently improves seedling growth and plant development (Herrera et al., 2016; Verma and White, 2017; Verma et al., 2017; Molina et al., 2021), suggesting that organelles are not damaged with the sterilization treatment. We agree with previous conclusions (Truyens et al., 2015; Herrera et al., 2016; Verma and White, 2017; Verma et al., 2017; Chang et al., 2021) that bacterial endomicrobiota of seeds are essential to seed germination and plant development.

### Core Bacterial Endophytes of *Jasione*

Most studies of plant microbiota are focused on the rhizosphere microbiome (Thijs et al., 2016; Mohanram and Kumar, 2019) because it is a reservoir of potential microorganisms with benefits for plants and therefore may be used as biofertilizers for sustainable agriculture. However, not all microorganisms from the rhizosphere are able to colonize efficiently into plant tissues; only 2–5% are considered plant growth-promoting bacteria (PGPB) (Antoun and Kloepper, 2001). Therefore, a better starting point to obtain plant microbes would be to focus on the study of the seed endomicrobiota (Matsumoto et al., 2021; Moreira et al., 2021); this especially since they are selected generation to generation for the ability to enter the host plant (Hardoim et al., 2008). Nevertheless, seeds have already shown to be a microbiota reservoir providing beneficial traits to the host plant (Ewald, 1987; Rudgers et al., 2009; Hardoim, 2019; Wang et al., 2021). *Jasione* persistent OTUs were genera *Pseudomonas* spp., *Ralstonia* spp., *Undibacterium* spp., *Cutibacterium* spp., and *Kocuria* spp. and family *Comamonadaceae*. These OTUs were inside all seedling plants of *J. montana* and *J. sessiliflora* and detected across all treatments. These OTUs have been previously identified as plant microbiota. Members of genus *Pseudomonas* have shown various PGP traits such as IAA and siderophore production and phosphate solubilization (Kumar et al., 2020). In addition, *Pseudomonas* spp. are common plant endophytes (Compant et al., 2011; Pacifico et al., 2019; Wang et al., 2021), acting also as biocontrol agents (Munakata et al., 2020) and members of the core microbiome of many plant species, including canola (*Brassica napus*) (Moreira et al., 2021). Recently, *Pseudomonas* has been found in rice plants with the ability to decrease arsenic translocation from roots into grains (Dolphen and Thiravetyan, 2019; Wang et al., 2021), and in lettuce roots as rare microbiota that protect against plant pathogens (Cardinale et al., 2015). Accordingly, other PGPB species, including *Cutibacterium* spp., identified in wheat (*Triticum*) seeds (Kuźniar et al., 2020; Abdullaeva et al., 2020); *Undibacteriumin* spp. identified in maize (*Zea mays*) (Liu et al., 2013); and *Kocuria* spp. identified in alfalfa (*Medicago sativa*) (López et al., 2018). Species of *Comamonadaceae* family were found to dominate barley (*Hordeum vulgare*) roots and rhizospheres (Bulgarelli et al., 2015). Species of genus *Ralstonia* can also colonize plants as endophytes (van Overbeek et al., 2004) and can be a diazotrophic endophyte with a capacity to increase N availability to plants (Patel and Archana, 2017). Together with species of genus *Pseudomonas*, *Ralstonia* species are part of the core microbiota of some grapevines (Pacifico et al., 2019). More recently, *Ralstonia eutropha* was found to alleviate As toxicity to wheat plants by increasing the efficiency of root energy metabolism and cell wall biosynthesis (Wang et al., 2018). Therefore, these OTUs can be considered as core bacterial endophytes since they are inside tissues of *J. montana* and *J. sessiliflora* and provide benefits to the host.

### Rare Endomicrobiota of Seeds

The long tail of the rank abundance curve (Figure 7) represents the rare microbiota of *Jasione*. Most of the OTUs were below 1% of the contribution to total diversity. Only a few OTUs such as *R. rhodochrous* (inoculated), *Sphingomonas* spp., and *Paenibacillus* spp. represent more than 50% of the total microbiota. For instance, the members of the genera associated with BA treatment such as *Asinibacterium* and *Nocardioides* are considered rare since they only represent less than 1% of the total diversity of the *Jasione* seed endomicrobiota. However, species of genus *Asinibacterium* have shown tolerance to uranium (Brzoska et al., 2019) and species of genus *Nocardioides* have shown halotolerance and methylotrophic strains with the capacity to alleviate salinity stress of crop plants (Meena et al., 2020). Species of genus *Brevundimonas*, associated with As treatment, only represented 0.4% but some strains showed versatility to degrade recalcitrant aromatic compounds (Parthasarathy et al., 2017). Thus, most of the OTUs from *Jasione* seeds were rare microbiota. However, despite their low contribution, they represent a large bacterial reservoir with rapid responses to environmental changes (Du et al., 2020; Ji et al., 2020), playing important physiological and ecological roles in improving host traits (Sogin et al., 2006; Elshahed et al., 2008; Lynch and Neufeld, 2015).

### Biotic and Abiotic Factors Controlling Endomicrobiota Structure

The degree of similarity between seed endomicrobiota of *J. montana* and *J. sessiliflora* is high. Endomicrobiota structure is a consequence of ecological and evolutionary forces driving microbial diversity and structure (West et al., 2019; Özkurt et al., 2020). The plant microbiome is thought to be controlled by various processes such as selection and speciation (which are deterministic events), drift, and dispersal represented by stochastic events (Vellend, 2016). Both niche (deterministic processes) and neutral (stochastic processes) theories have been advanced to explain microbial community assembly (Zhou and Ning, 2017; Wilber et al., 2020). Previous works have shown (Wilber et al., 2020) that abiotic (i.e., As) and biotic (i.e., addition of *R. rhodochrous*) factors affect bacterial selection from the host microbial assemblage after accounting for the effects of dispersal and drift.

The high similarity between the seed endomicrobiota of both species and the fact that *J. montana* and *J. sessiliflora* are phylogenetically very close could be a starting point to investigate coevolution between these two organisms. *Jasione* microbial assemblage are modulated by abiotic (i.e., As) or biotic (i.e., addition of *R. rhodochrous*) factors with important consequences at a functional and genetic level. From a functional point of view, these closely related species (plant genome plus its associated microbiota) showed different responses against stresses. The negative consequences of the As stress were only observed within *J. sessiliflora* but not in *J. montana*. These results would suggest that only *J. montana* have physiological traits against As stress. The exclusive *J. montana* core bacterial endophyte was composed by genus *Rhodococcus.* Members of this genus (*R. aetherivorans*, *R. erythropolis*, *R. equi*, and *R. rhodochrous*) have also been described as PGPB or endophytes resistant to As (Gu et al., 2018; Molina et al., 2021; Navazas et al., 2021). From a functional point of view, *R. rhodochrous* has been shown to colonize efficiently *Jasione* species, modifying the response to As stress. According to Thijs et al. (2016), plant microbiota assembly under environmental stress depends on the recruitment of key species. The bioaugmentation with *R. rhodochrous* provides different physiological traits depending on the *Jasione* species. For instance, *R. rhodochrous* provided synergistic mechanisms to *J. montana*, increasing the number of leaves. The same endophyte was antagonistic to *J. sessiliflora*, decreasing the number of LR. It is interesting that under As stress, *R. rhodochrous* provided benefits to both species, increasing the number of LR over the control conditions under equivalent stress. Previous investigations have already shown that continuous exposure of plants to stress conditions decreases plant endogenous IAA levels. However, the inoculation with endophytes and rhizobacteria increases IAA synthesis (Sheng et al., 2008; Rajkumar et al., 2009; Khaksar et al., 2017). Therefore, *R. rhodochrous* may affect *Jasione* traits in two ways as follows. *R. rhodochrous* is a PGPB that is resistant to As and produces IAA (Molina et al., 2021). This phytohormone increases LR (Lavenus et al., 2013) but also alleviates ROS accumulation produced by As stress (Khaksar et al., 2017). In addition, *R. rhodochrous* is capable of metabolizing As (V) to organic forms, possibly less toxic (Molina et al., 2021), which is an obvious advantage for both species but especially for *J. sessiliflora*, which does not have this microorganism among the components of its microbiota. Therefore, *R. rhodochrous* may modulate *Jasione* root plasticity, which is a key adaptative trait to cope As stress (Lavenus et al., 2013). Our results provide empirical evidence for the role of a key species (*R. rhodochrous*) transmitted horizontally, driving the structure and final functionality of host endomicrobiota against As stress. Our findings suggest the *Jasione* traits are influenced by *R. rhodochrous* and associated microbiota (Ravanbakhsh et al., 2019) and improve host response to As stress.

## Conclusion

In this study, we have shown that *Jasione* traits are influenced by both *R. rhodochrous* and seed-associated endomicrobiota and improve host response against As stress. Our experimental evidence shows that *R. rhodochrous* efficiently colonizes *Jasione* plants, influencing the structure and final functionality of the endomicrobiota. Furthermore, in this study, next-generation sequencing technologies (NGS-Illumina) helped to identify *Jasione* core bacterial endophytes composed of genera *Pseudomonas* spp., *Ralstonia* spp., *Undibacterium* spp., *Cutibacterium* spp., and *Kocuria* spp. and family Comamanadaceae. This technology also provided empirical evidence of the rare endomicrobiota inside *Jasione* seeds. These rare members represent most of the total diversity, and despite their low contribution, they offer important physiological and ecological traits to host. Therefore, plant seed endomicrobiota or endophytes should be considered to be a resource for exploitation; they have potential applications and utility to environmental remediation, sustainable agriculture, and biotechnology.

## Data Availability Statement

The datasets presented in this study can be found in online repositories. The names of the repository/repositories and accession number(s) can be found below: https://www.ncbi.nlm.nih.gov/, SUB9031392. Sequence data were deposited in the NCBI Sequence REacd Archive, Bioproject ID PRJNA699582, accessions SAMN17799125 to SAMN17799134.

## Author Contributions

MM and NG-B: conceptualization and methodology. IM-R, IC, and MA: the expertise on the software. NG-B, IM-R, and MA: formal analysis. NG-B and IM-R: writing the original draft. JW: showing the techniques to manipulate and isolate endophytes. MM, MA, IC, and JW: review and editing. All authors contributed extensively to the work, and read and agreed to the published version of the manuscript.

## Conflict of Interest

The authors declare that the research was conducted in the absence of any commercial or financial relationships that could be construed as a potential conflict of interest.

## Publisher’s Note

All claims expressed in this article are solely those of the authors and do not necessarily represent those of their affiliated organizations, or those of the publisher, the editors and the reviewers. Any product that may be evaluated in this article, or claim that may be made by its manufacturer, is not guaranteed or endorsed by the publisher.

## Footnotes

1
https://imagej.nih.gov/ij/

2
https://data.qiime2.org/2020.6/common/silva-138-99-515-806-nb-classifier.qza
