# Supplementary figures and images for: Endophytic Microbes Are Tools to Increase Tolerance in Jasione Plants Against Arsenic Stress
Source: Front Microbiol. 2021 Oct 6;12:664271. doi: 10.3389/fmicb.2021.664271 (PMC8527096; doi:10.3389/fmicb.2021.664271)

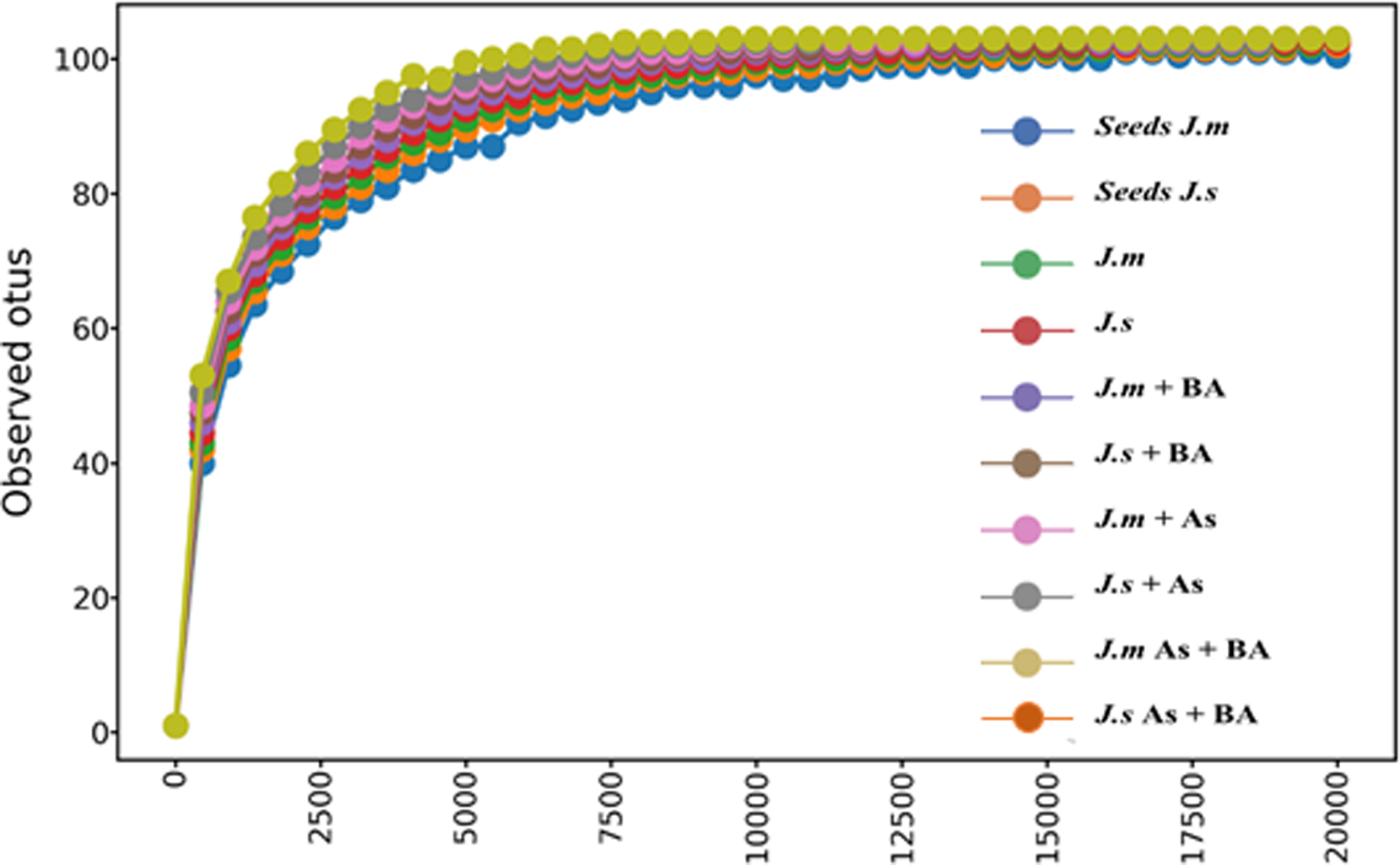

Supplement: Supplementary file 1 [file Image_1.TIF]
